# Photoacoustic imaging of posterior periodontal pocket using a commercial hockey-stick transducer

**DOI:** 10.1117/1.JBO.27.5.056005

**Published:** 2022-05-24

**Authors:** Lei Fu, Chen Ling, Zhicheng Jin, Jessica Luo, Jorge Palma-Chavez, Zhuohong Wu, Jingcheng Zhou, Jiajing Zhou, Brian Donovan, Baiyan Qi, Aditya Mishra, Tengyu He, Jesse V. Jokerst

**Affiliations:** a University of California San Diego, Department of NanoEngineering, La Jolla, California, United States; b University of California San Diego, Materials Science and Engineering Program, La Jolla, California, United States; c University of California San Diego, Department of Radiology, La Jolla, California, United States

**Keywords:** photoacoustic imaging, periodontitis, clinical attachment loss, oral health, optoacoustics

## Abstract

**Significance:**

Photoacoustic imaging has shown advantages over the periodontal probing method in measuring the periodontal probing depth, but the large size of conventional photoacoustic transducers prevents imaging of the more posterior teeth.

**Aim:**

Our aim is to develop a photoacoustic imaging system to image the more posterior periodontal pocket.

**Approach:**

We report a clinical “hockey-stick”-style transducer integrated with fibers for periodontal photoacoustic imaging. Cuttlefish ink labeled the periodontal pocket as the photoacoustic contrast agent.

**Results:**

We characterized the imaging system and then measured the pocket depth of 35 swine teeth. Three raters evaluated the performance of the hockey-stick transducer. The measurements between the Williams probing (gold standard) and the photoacoustic methods were blinded but highly correlated. We showed a bias of ∼0.3  mm for the imaging-based technique versus Williams probing. The minimum inter-reliability was over 0.60 for three different raters of varying experience, suggesting that this approach to measure the periodontal pocket is reproducible. Finally, we imaged three pre-molars of a human subject. We could access more upper and posterior teeth than conventional linear transducers.

**Conclusions:**

The unique angle shape of the hockey-stick transducer allows it to image more posterior teeth than regular linear transducers. This study demonstrated the ability of a hockey-stick transducer to measure the periodontal pocket via photoacoustic imaging.

## 1 Introduction

Periodontitis remains a significant public health concern. According to the Office of Disease Prevention and Health Promotion, 37.4% of adults aged 45 to 74 years had moderate or severe periodontitis in 2013 to 2014.^1^ Periodontitis has been linked to diabetes,^2^ infective endocarditis,^3^ cardiovascular disease,^4^ respiratory disease,^5^ and mental illness.^6^ Periodontitis is a chronic inflammatory disease caused by subgingival bacteria that destroy the teeth’s supporting structures.^7^ Periodontitis can lead to gingival inflammation, gingival recession, bone mobility, and bone/tooth loss.^8^ The extent of periodontitis is measured to indicate the treatment and monitor the treatment response. Bone loss can be measured on radiographs,^9^ and mobility is measured via manual manipulation.^10^ The degree of inflammation is subjective and is based on the color of the gingiva and the frequency of bleeding on probing.^11^ Recession is the main characteristic of periodontitis and is associated with clinical attachment loss (CAL).^12^ CAL is assessed by measuring the gingival margin (GM) and the pocket depth. Oral health professionals generally use a periodontal probe such as a Williams probe to measure the pocket depth.^13^ However, periodontal probing can be affected by the probing force, the insertion point, and the probing angulation.^14^^,^^15^ Periodontal probing can also cause bleeding, is painful to the patient, and is time consuming for the provider.

Dental ultrasound imaging is increasingly used to characterize both hard and soft tissues in the oral cavity.^16^^,^^17^ Ultrasound imaging has been used for dental implant studies,^18^^,^^19^ and high-resolution ultrasound imaging can help identify the gingival sulcus, GM, alveolar bone, alveolar bone crest, and the cementoenamel junction.^20^^–^^22^ One subset of ultrasound is photoacoustic imaging, which creates images via the ultrasound created by incident light pulses.^23^^,^^24^ We previously showed that photoacoustic-ultrasound imaging can measure the periodontal pocket with a food grade contrast agent (cuttlefish ink) in swine and humans with good correlation to standard probing.^25^^,^^26^ However, the fundamental limitation of the existing technology is the relatively large size of the transducers, which prevents imaging molars and pre-molars. Although there are miniaturized transducers for anorectal,^27^ endocranial,^28^ and laparoscopic imaging,^29^ these systems often operate at a central frequency (5 to 7 MHz) and do not have sufficient resolution to image the oral anatomy.

Here, we added laser excitation to a so-called hockey stick transducer for photoacoustic imaging of the periodontal pocket. We were motivated to use this commercially available transducer because of its relatively high bandwidth (7 to 15 MHz) and angled design suitable for imaging the posterior of the mouth.^30^^,^^31^ Although designed for superficial applications such as intraoperative neural imaging and musculoskeletal imaging,^32^ the hockey-stick design is also suitable for imaging the molars (up to the first molar in human subjects). Our work here integrated optical fibers for both photoacoustic and ultrasound imaging. We then characterized the performance of the system with tissue-mimicking phantoms and *ex vivo* porcine jaws. Finally, we showed that we could image pre-molars #5, #12, and #29 in a healthy human subject.

## 2 Materials and Methods

### 2.1 Hardware

A commercially available hockey-stick transducer (ATL CL15-7, Philips) received the photoacoustic signal with a central frequency of 9 MHz and bandwidth of 7 to 15 MHz. We used a 14-fiber bundle to repurpose the transducer for photoacoustic imaging (2.5-mm-diameter fibers). These fibers were coupled to a tunable optical parametric oscillator (OPO) laser operating at 680 to 970 nm (OPOTek). A customized holder was 3D printed to mount the fiber bundles on the transducer at the desired illumination angle of 45 deg. The pulsed energy from the fiber bundle is 22  mJ/pulse with 5 to 7 ns light pulses. A research ultrasound data acquisition system (Vantage; Verasonics, Inc., Kirkland, Washington, United States) was used to collect and preprocess photoacoustic signals. The Vantage system has 256 channels with a sampling rate of 62.5 MHz. The frame rate is 20 Hz.

### 2.2 *Ex Vivo* Validation

We used a pencil lead array to evaluate the light focus. Twelve pencil leads (0.2 mm diameter) were put into a 3D-printed holder that keeps them parallel and 2 mm apart.^33^ Swine jaws were obtained from a local abattoir. The pocket depths of natural swine jaws are below 2 mm, but periodontal pockets are usually deeper than 5 mm in humans.^34^ Thus, we made artificially deep pockets ranging from 3 to 7 mm with a scalpel; 35 swine teeth were prepared including 15 artificial pockets. The melanin nanoparticles in cuttlefish ink (Nortindal, Spain) were used as the photoacoustic contrast agent to highlight the pocket.^25^^,^^26^ The absorption and photoacoustic spectra of the cuttlefish melanin particles were also measured previously and shown to be relatively flat absorbers.^25^^,^^26^

### 2.3 Pocket Depth Measurement

Pocket depth measurements were blinded between the Williams probing method and photoacoustic method. The Williams probe has black ring markings at 1, 2, 3, 5, 7, 9, and 10 mm from the tip and was placed into the pocket along the direction of the tooth root.^16^ The photoacoustic images were collected along with ultrasound data for anatomy. The ultrasound and photoacoustic images were collected at the same time and comprised the same imaging area. These two images were then synchronized in ImageJ.^35^ The photoacoustic image shows the distribution of the cuttlefish ink and was used to measure pocket depth: the distance from the GM on ultrasound to the farthest extent of cuttlefish ink in the pocket by photoacoustic imaging. Three raters with different experiences rated the images. Rater 1 (LF) has 1 year of experience in analyzing periodontal images. Rater 2 (JL) has 1 month in analyzing the periodontal images. Rater 3 (JZ) was trained by Rater 1 for 30 min prior to making measurements.

### 2.4 Inter-Rater Reliability

We used the inter-rater reliability (IRR) to qualify the agreement between each two of the three raters via inter-class correlation (ICC).^36^ We calculated ICC in case ICC (2,1); here, the “2” means that several judges are selected and each judge measures all of the pocket depths, and 1 stands for the reliability of a single rater.^37^

### 2.5 Human Subjects Imaging

We recruited a healthy 29-year-old adult with good oral hygiene. All work with human subjects was approved by the UCSD institutional review board (IRB) and conducted according to the ethical standards set forth by the IRB and the Helsinki Declaration of 1975. The participant gave written informed consent, and incisors and pre-molars were imaged. Both the volunteer and operator wore near-infrared protective laser goggles during the experiments. The cuttlefish ink contrast agent for the human subject was prepared as described.^25^ We used water as the diluent. An ultrasound gel pad (Parker laboratories, Inc., United States) was placed inside a sterile sleeve for coupling to ensure sufficient offset between the transducer and tissue. Ultrasound gel was also placed inside the sleeve for human studies. Water was used on the exterior of the sleeve to facilitate coupling to the tissue.

## 3 Results and Discussion

We first adjusted the hockey-stick transducer to be suitable for photoacoustic imaging. We describe the performance metrics of that transducer and strategies to couple optical excitation, and then we validate swine *ex vivo* and human subjects *in vivo*.

### 3.1 Hockey-Stick Transducer

**Fig. 1:**
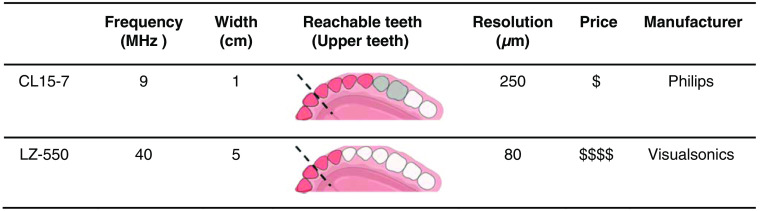
Performance metrics of the two transducers: CL15-7 and LZ-550. Reachable teeth in photoacoustic imaging are marked in red. Reachable teeth in ultrasound are marked in gray. The dashed line is the midline of human teeth.

Figure 1 compares the two different transducers suitable for periodontal imaging including a 9 MHz hockey-stick transducer (CL15-7) and a 40 MHz transducer (LZ-550).^25^^,^^26^ The two transducers have different shapes. The hockey-stick transducer has a width that is roughly fivefold smaller than the conventional transducer, and the cost is much less than the LZ-550. The LZ-550 has a higher resolution (80  μm) than the CL15-7 (250  μm). Our goal was to image teeth in the posterior of the mouth. The gray teeth represent those that are accessible for ultrasound imaging, and the red ones represent those accessible for photoacoustic-ultrasound imaging. The hockey-stick transducer can image the upper incisors, cuspids, and the first pre-molar; the LZ-550 can only image the upper incisors in photoacoustic mode.

### 3.2 Integration of Optical Components for Photoacoustic Imaging

**Fig. 2:**
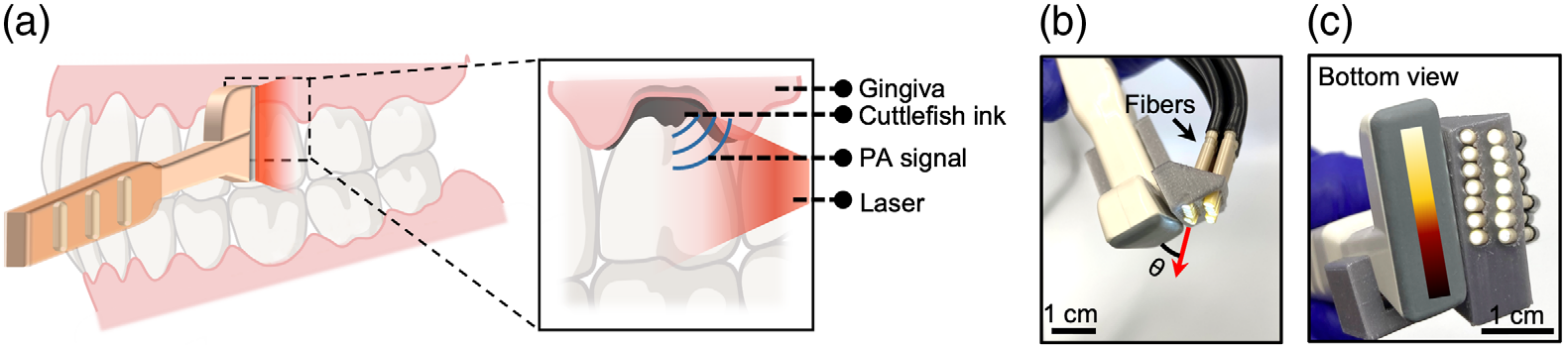
Transducer evaluation. (a) Photoacoustic imaging detects the periodontal pocket with a hockey-stick transducer. The inset shows that cuttlefish ink to highlight the pocket. The contrast agent labels the periodontal pocket underneath the gingiva for photoacoustic imaging. (b) and (c) Hockey-stick transducer with fiber bundle from front view and bottom view, respectively. One side of the transducer is arranged with two rows of fibers while the other side has no fibers because it needs to fit into the oral cavity.

Figure 2(a) shows the hockey-stick transducer, illustrating how it contacts the surface of tooth and gingiva. The transducer surface faces the sagittal tooth surface and gingiva, and the handle projects out toward the operators. We used the CL 15-7 to obtain ultrasound images of the first molars (#3, #14, #19, and #30) in a human subject. The periodontal pocket is usually less than 5 mm below the GM, which fits the near field metric of a hockey-stick transducer.

Figures 2(b) and 2(c) show a front view and bottom view of the hockey-stick transducer with the fiber bundle. We used 14 fibers in two rows to deliver a pulse laser to the top of the transducer. Our imaging target is at the location ∼1  cm surrounding the GM, and thus we only needed to use half of the 128 elements in the 2-cm transducer. The angle of fiber orientation was 45 deg and was chosen based on the acoustic focal zone (5 to 10 mm from the transducer) and the offset required for the coupling pad. Unfortunately, integration of the fibers added several cubic centimeters of bulk to the transducer. One side of the transducer is arranged with two rows of fibers while the other side has no fibers because it needs to fit into the oral cavity [Fig. 2(b)].

### 3.3 Characterization

**Fig. 3:**
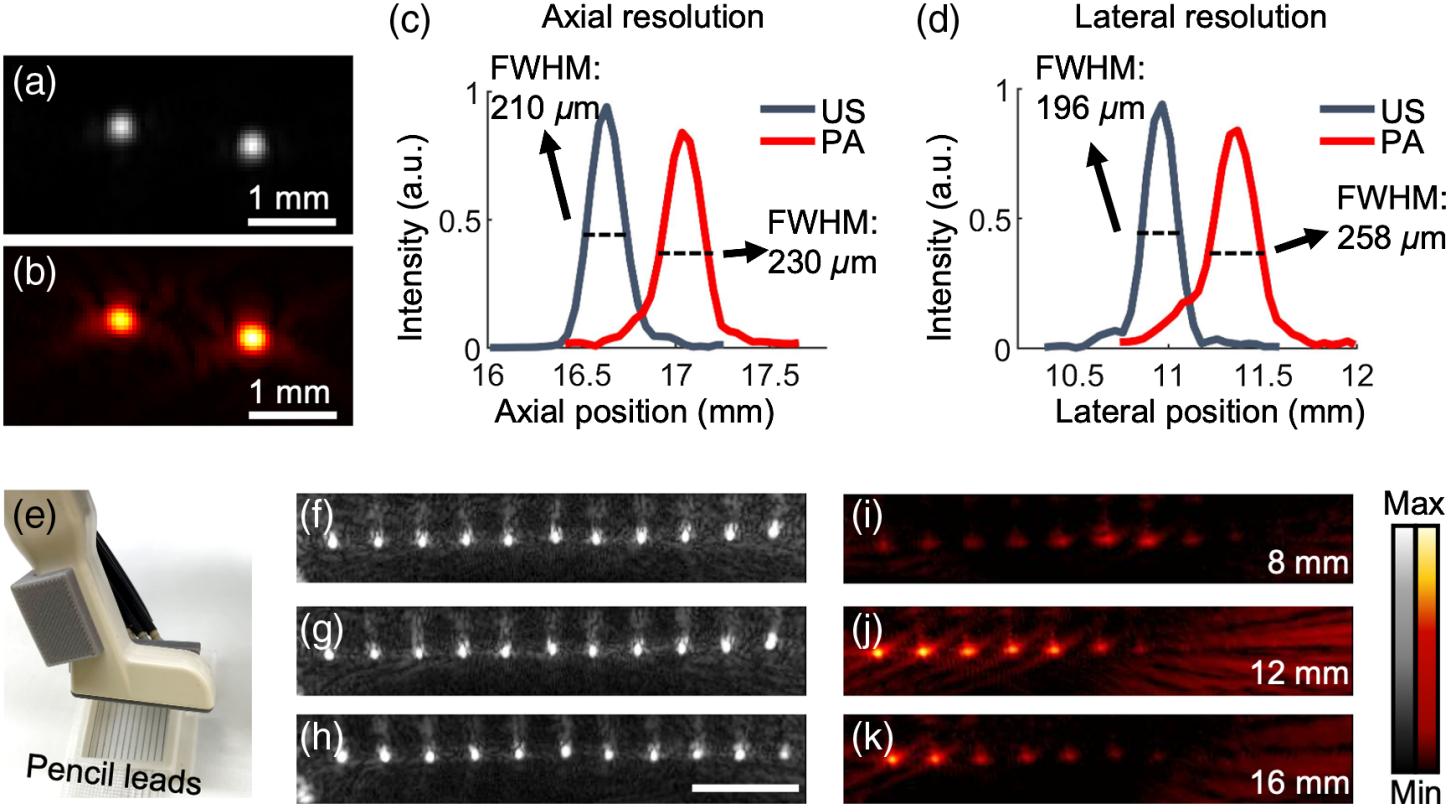
Performance metrics of customized transducer. (a) Ultrasound image of the cross-section of two nichrome wires. (b) Photoacoustic image of the nichrome wires. (c) Axial ultrasound (US) and photoacoustic (PA) amplitude distributions along the left nichrome wire. 210 and 230  μm are the respective axial resolutions. (d) Lateral ultrasound and photoacoustic amplitude distribution along the left nichrome wire. 196 and 258  μm are respective lateral resolutions. (e) Schematic of pencil lead array imaging. Panels (f), (g), and (h) are the ultrasound images when the pencil leads array is 8, 12, and 16 mm below the transducer, respectively. Panels (i), (j), and (k) are the photoacoustic images when the pencil leads array is 8, 12, and 16 mm below the transducer, respectively. The scale bar is 5 mm.

We characterized the resolution and the imaging range of the system. The lateral and axial resolutions were determined by imaging the cross-section of two nichrome wires with a diameter of 30  μm. An ultrasound image of the wire cross-sections is shown in Fig. 3(a), and the photoacoustic image is shown in Fig. 3(b). The lateral and axial amplitude distributions across the left nichrome wire were extracted from Figs. 3(a) and 3(b); the axial data are shown in Fig. 3(c), and the lateral data are shown in Fig. 3(d). We define the full width at half maximum of the lateral and axial amplitude distributions as the axial and lateral resolutions, respectively.^38^ The axial and lateral resolutions in photoacoustic imaging are 230 and 258  μm, respectively. The axial and lateral resolutions in ultrasound imaging are 210 and 196  μm, respectively.

We also imaged an array of pencil leads (12 leads, 24 mm wide) to examine the homogeneity of the incident fluence [Fig. 3(c)]. A stronger photoacoustic signal indicates a better light focus. The array was placed 8, 12, and 16 mm underneath the transducer as shown in the ultrasound images in Figs. 3(f)–3(h), correspondingly. Figures 3(i)–3(k) are the photoacoustic images. Obviously, the left half of the array has a stronger photoacoustic signal than the right half because we only need the left half of the array for periodontal imaging, and it is where the optical fibers are focused. The strongest photoacoustic signal is seen 12 mm beneath the transducer; this 12-mm depth should have the highest optical fluence based on the 45 deg angle used when coupling the optical fibers to the transducer.

### 3.4 Imaging the Williams Probe in the Periodontal Pocket

**Fig. 4:**
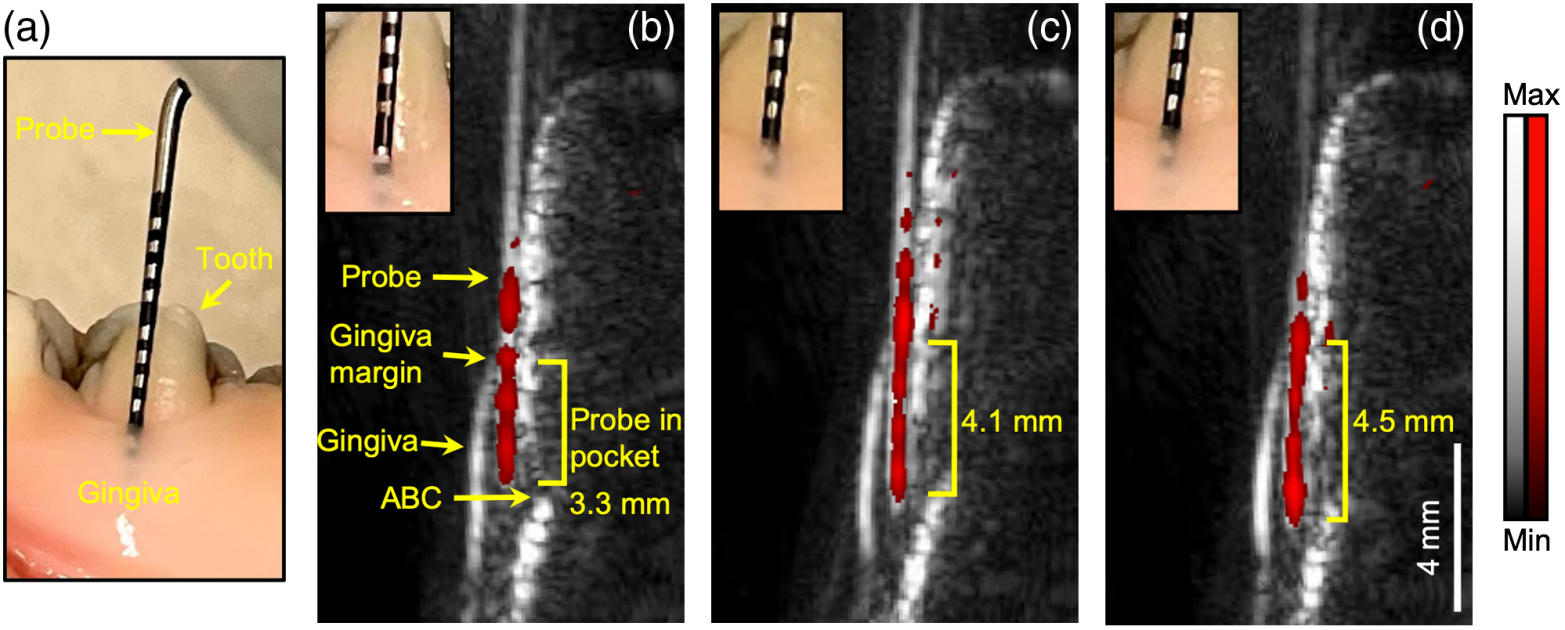
A Williams probe measures the pocket depth and offers photoacoustic contrast. (a) Williams probe in the periodontal pocket with 1-mm black tick marks on the probe. Panels (b), (c), and (d) are a stack of photoacoustic-ultrasound images when the probe is inserted into the pocket at probing depths of 3.3, 4.1, and 4.5 mm, respectively, with the hockey-stick transducer. The insets are zoomed photos of the probe in the pocket. Ultrasound image is in gray, and photoacoustic image is in red. The red line is the probe. ABC, gingiva, GM, and probe tip in the pocket are obvious.

We next evaluated the utility of the modified transducer to measure periodontal probing depths. The Williams probe reports depths in integer values to the nearest millimeter [Fig. 4(a)]. In contrast, image-based measurements have a resolution as high as 250  μm when using the hockey-stick transducer. Here, we evaluated distortions produced during photoacoustic imaging by imaging the Williams probe itself [Fig. 4(a)]. In other words, the Williams probe detects the pockets depth and works as a contrast for photoacoustic imaging at the same time. Figures 4(b)–4(d) show the photoacoustic-ultrasound images acquired by the hockey-stick transducer when the Williams probe was inserted into the pocket from around 3.0 to 4 mm. The structure of tooth is distinguishable in these images. We identify the Williams probe as a long red line in the photoacoustic image. Part of the Williams probe is in the pocket. Figures 4(b) and 4(d) show the GM and the probe tip. We measured the distance between the GM and the tip as the probe length in the pocket. The insets show the probe in the pocket when the photoacoustic-ultrasound image is acquired. We qualified the three probe lengths in Figs. 3(b)–3(d) by photoacoustic imaging with five replicates: 3.3, 4.1, and 4.5 mm, respectively. In comparison, the probing depths are 3, 4, and 4 mm by reading the Williams probe by eye, which is less precise than the photoacoustic method as the probe was being inserted deep into the pocket.

### 3.5 Periodontal Pocket Depth Measurements

#### 3.5.1 Pocket depth measurements in swine model

We used the swine tooth model to validate the performance of the hockey-stick transducer because swine teeth have structures similar to human teeth.^39^ We first used a Williams probe to measure the pocket depth and then performed photoacoustic imaging to measure the same pocket for comparison. Ultrasound/photoacoustic images were also acquired before applying the cuttlefish ink contrast agent as control images.

**Fig. 5:**
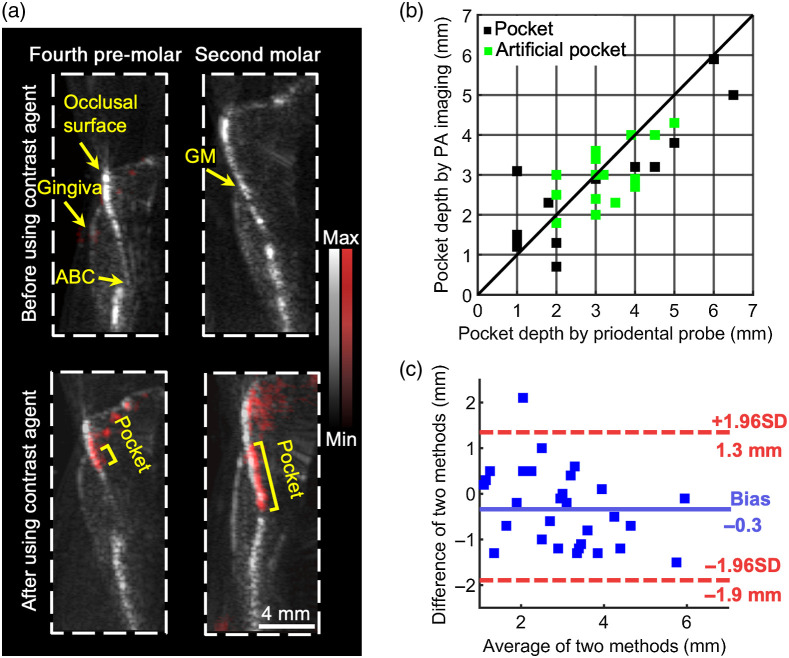
(a) Ultrasound/photoacoustic images of two swine teeth, the fourth pre-molar and the second molar in sagittal view. Photoacoustic data are in red. Ultrasound data are in gray. Images on the top are before administering the cuttlefish ink, and images on the bottom are after; hence, there is red signal. (b) Pocket depth measurements with 35 replicates. The x-axis represents the measurements with Williams probing, and the y-axis represents the measurements with photoacoustic imaging. Black data points are the natural pockets, and green data points are the artificial pockets. (c) Bland–Altman analysis of the statistics in (b). The blue line is the bias of photoacoustic measurements over probing measurements, which is −0.3  mm. The two red lines are the upper and lower LOA; 95% of the replicates fell into the region between the two lines.

Figure 5(a) shows the ultrasound/photoacoustic images of two swine teeth: the fourth pre-molar and the second molar. The photoacoustic image in red is overlaid with the ultrasound image in gray. The top two images in Fig. 5(a) show the gingiva, GM, and occlusal surface tooth and their associated tissue before applying the contrast agent. The alveolar bone and alveolar bone crest (ABC) are distinguishable in the fourth pre-molar.

The bottom panels in Fig. 5(a) were collected after the sulcus was irrigated with the cuttlefish ink. The contrast agent is shown as a line below the GM, i.e., the periodontal pocket. The probing depth can then be extracted from these images via image analysis from the bottom of the sulcus to the GM. The periodontal pocket is deeper in the second molar (5 mm) than in the fourth pre-molar (1.2 mm).

We then imaged 35 swine teeth to further evaluate the analytical utility of image-based pocket depth measurements. Three different raters independently measured the pocket depth. The x-axis represents the Williams probing method, and the y-axis represents the photoacoustic method. Black data points are the natural pockets and green data points represent the artificial pockets (see Sec. 2.2). The differences of the two measurements are much less than 1 mm, and most data are close to the convergent line. Figure 5(c) is the Bland–Altman analysis of Fig. 5(b), which qualifies the agreement between the photoacoustic and the Williams probing methods. It shows that 95% of the replicates fell within 3.2 mm of the differences between the two methods^40^; the bias is −0.3  mm. The bias can be understood as, when the pocket depth goes deeper, the contrast agent cannot penetrate through as these photoacoustic measurements are less than the probing measurements as shown in Fig. 5(b).^26^^,^^41^

Table 1 shows the Bland–Altman analysis of the three raters. Rater 2 has the smallest bias of −0.06  mm, whereas Rater 1 has the smallest lower limits of agreements (LOA). The negative bias was consistent across the three raters. We quantified the agreement level between each two of the three raters by IRR, which is estimated by calculating the ICC (see Methods). As a rule of thumb, Cicchetti (1994) provides commonly-cited cutoffs for qualitative ratings of agreement based on ICC values with IRR being poor for ICC values less than 0.40, fair for values between 0.40 and 0.59, good for values between 0.60 and 0.74, and excellent for values between 0.75 and 1.0.^36^ Table 2 shows the ICC between Raters 1 and 2, Raters 1 and 3, and Raters 2 and 3. The agreement between Raters 1 and 2 is 0.9 (excellent). The agreements between Raters 1 and 3 as well as Raters 2 and 3 are 0.64 (good) and 0.61 (good), respectively. The differences of agreement make sense because Rater 3 had been trained by Rater 1 for only 30 min. Similar to clinical ultrasound imaging, interpreting the photoacoustic/ultrasound images of tooth and the associated structure requires specific expertise and is a major challenge. Previously, the ICC for dental students was 0.51 (95% CI: 0.33 to 0.75) and 0.41 (95% CI: 0.24 to 0.64) for dental faculty.^42^ Our value reaches a clinical significance level that is shown to be at least at a “good,” suggesting that our newly developed method is systematic and teachable. The definitions of excellent and good are given in Sec. 2.4.

**Table 1:** Bland–Altman analysis of the three raters. The three raters measured the 35 pocket depths independently (by imaging). The values from the Williams probe were constant and were measured by an investigator blinded to the imaging results. Rater 1 has 1-year experience in photoacoustic periodontal imaging. Rater 2 has 1-month experience in photoacoustic periodontal imaging. Rater 2 was trained by Rater 1 for 30 min before doing measurements.

Bland–Altman analysis			
	Rater 1	Rater 2	Rater 3
Bias (mm)	−0.3	−0.06	−0.25
+1.96 SD (mm)	1.3	1.85	1.93
–1.96 SD (mm)	–1.9	–1.98	–2.43

**Table 2:** Inter-rater reliability between each two of the three raters. Rater 3 has the least experience in analyzing the photoacoustic/ultrasound images.

Inter-rater reliability			
Raters	Raters 1 and 2	Raters 1 and 3	Raters 2 and 3
ICC	0.90	0.64	0.61

#### 3.5.2 Pocket detection in a human subject

We also used the hockey-stick transducer to image the pocket of a healthy human subject. The frame rate is 20 Hz, which can help solve issues related to subject motion and probe stabilization. Since human oral is a hydrated environment, we used a very little amount of water instead of ultrasound gel to couple the transducer to the teeth and gums, which improved the clinical experience while providing the same imaging quality as using ultrasound gel.

**Fig. 6:**
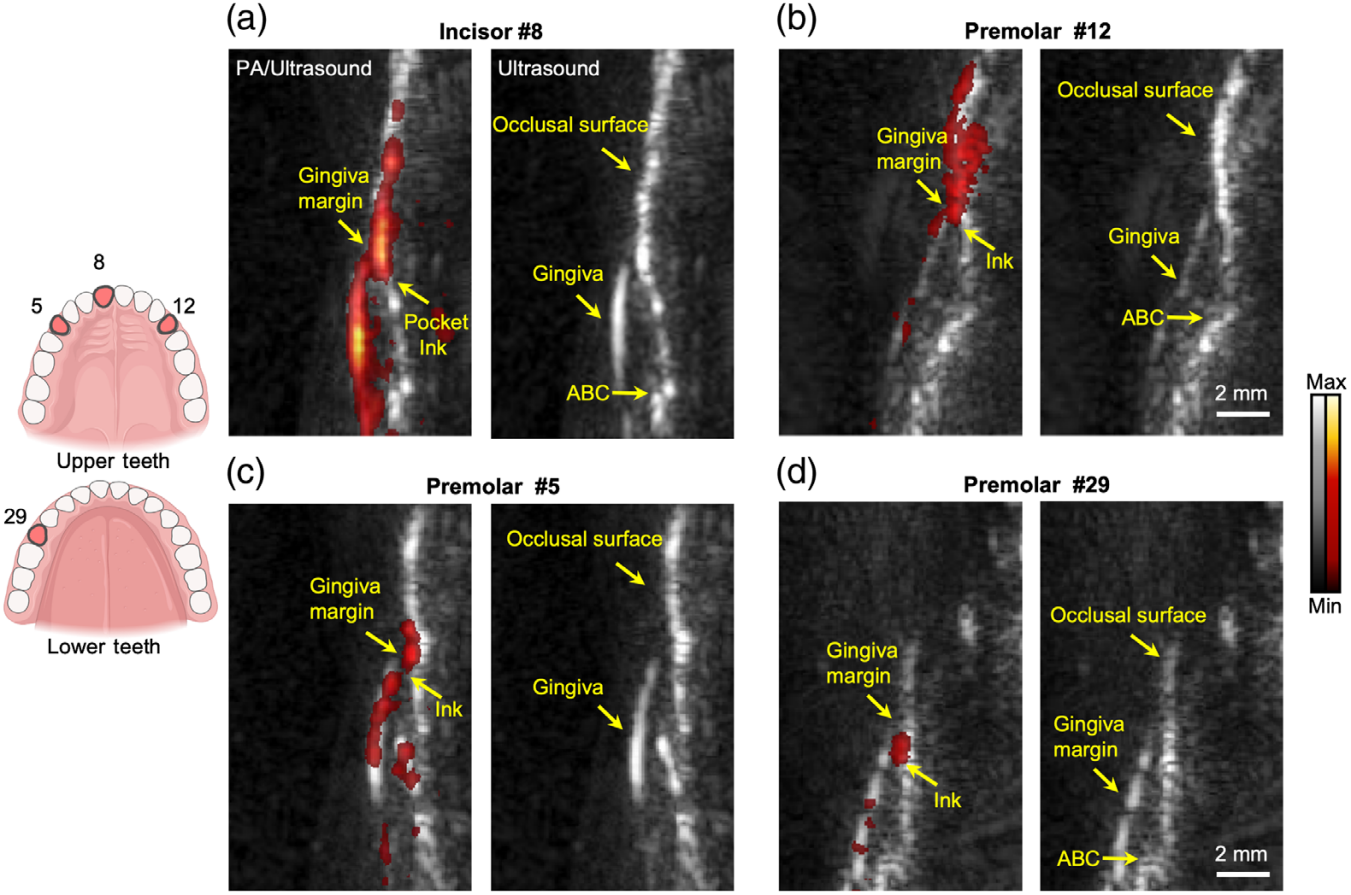
Photoacoustic/ultrasound images of four human teeth: (a) incisor #8, (b) pre-molar #12, (c) pre-molar #5, and (d) pre-molar #29. Cuttlefish ink contrast agent labels the periodontal pockets (left panels). Photoacoustic images in red are overlaid to the ultrasound images in gray. Right panels are the ultrasound images, correspondingly. Ultrasound images show the common structure of the tooth. Photoacoustic images show the position of the contrast agent.

We imaged four teeth of a healthy human subject: incisor #8, pre-molar #12, pre-molar #5, and pre-molar #29 as shown in Fig. 6. The contrast agent was administered to the teeth. The left panels show the photoacoustic-ultrasound overlaid images, and the right panels show the ultrasound images. All of the images are in sagittal view. The ultrasound images show that human teeth have structures similar to swine teeth. The gingiva, occlusal surface, and pocket are distinguishable in the ultrasound images. Photoacoustic/ultrasound images in the left panels show that the contrast agent labels the pocket as well as the gingiva and occlusal surface, where the periodontal pockets and the GMs can be identified in the photoacoustic images. We measured the pocket depths as (a) 0.9, (b) 0.5, (c) 0.3, and (d) 0.7 mm which are close to the results of a healthy human subject.^25^ Healthy subjects generally have much lower pocket depths than periodontal cases.

This hockey-stick transducer still has some limitations. First, the last two molars of human subjects are not imageable in photoacoustic mode due to the large size of the device. The illumination angle from the fibers requires around a 1.0 cm gap between the transducer and the tissue for light coupling. This gap and the size of the fiber module prevent the transducer from covering all of the teeth. Second, high-frequency transducers with smaller dimensions that better fit the human oral cavity are needed. The periodontal imaging depth (gingival thickness) is less than 5 mm, which allows for use of a high-frequency transducer. The periodontal pocket can be as deep as 10 mm. However, current commercial high-frequency transducer can hardly image pre-molars and molars of a human subject. Thus, an even smaller linear array could be developed and still cover all teeth in clinical studies.

## 4 Conclusion

The unique angle shape of the hockey-stick transducer allows it to image the more posterior teeth than regular linear transducers. This study mainly demonstrated the ability of hockey-stick transducer to detect the periodontal pocket in photoacoustic imaging. The measurements using the photoacoustic and Williams probes agree well with each other in the swine model. We used the hockey-stick transducer to image incisor #8 and pre-molars #5, #12, and #29 of a healthy human subject. We envision a more compact high-frequency transducer that is designed specifically for dental imaging to cover all of the teeth of any human subject.

## Code, Data, and Materials Availability

Data can be requested from the authors at any time.

## References

[1] Centers for Disease Control and Prevention, National Center for Health Statistics (CDC/NCHS), National Health and Nutrition Examination Survey (NHANES), “Adults with moderate or severe periodontitis (percent, 45–74 years),” https://www.healthypeople.gov/2020/data/Chart/5026?category=1&by=Total&fips=-1.

[2] Preshaw P. M. et al., “Periodontitis and diabetes: a two-way relationship,” Diabetologia 55(1), 21–31 (2012). DBTGAJ 0012-186X 10.1007/s00125-011-2342-y 22057194 PMC3228943

[3] Ninomiya M. et al., “Relationship of oral conditions to the incidence of infective endocarditis in periodontitis patients with valvular heart disease: a cross-sectional study,” Clin. Oral Invest. 24(2), 833–840 (2020). 10.1007/s00784-019-02973-2 31197658

[4] Seymour G. J. et al., “Infection or inflammation: the link between periodontal and cardiovascular diseases,” Future Cardiol. 5(1), 5–9 (2009). 10.2217/14796678.5.1.5 19371196

[5] Bansal M. Khatri M. Taneja V., “Potential role of periodontal infection in respiratory diseases—a review,” J. Med. Life 6(3), 244–8 (2013). 24155782 PMC3786481

[6] Genco R. J. et al., “Relationship of stress, distress, and inadequate coping behaviors to periodontal disease,” J. Periodontol. 70(7), 711–723 (1999). 10.1902/jop.1999.70.7.711 10440631

[7] Mariotti A. Hefti A. F., “Defining periodontal health,” BMC Oral Health 2015, S6 (2015). 10.1186/1472-6831-15-S1-S6 PMC4580771 26390888

[8] Highfield J., “Diagnosis and classification of periodontal disease,” Aust. Dent. J. 54, S11–S26 (2009). ADEJA2 0045-0421 10.1111/j.1834-7819.2009.01140.x 19737262

[9] Vidor M. M. et al., “Imaging evaluating of the implant/bone interface—an *in vitro* radiographic study,” Dentomaxillofac. Rad. 46(5), 20160296 (2017). 10.1259/dmfr.20160296 PMC5595032 28211288

[10] Aminoshariae A. et al., “Declassifying mobility classification,” J. Endodont. 46(11), 1539–1544 (2020). 10.1016/j.joen.2020.07.030 32768419

[11] Caton J. Greenstein G. Polson A. M., “Depth of periodontal probe penetration related to clinical and histologic signs of gingival inflammation,” J. Periodontol. 52(10), 626–629 (1981). 10.1902/jop.1981.52.10.626 6975365

[12] Farook F. F. et al., “Reliability assessment between clinical attachment loss and alveolar bone level in dental radiographs,” Clin. Exp. Dent. Res. 6(6), 596–601 (2020). 10.1002/cre2.324 32918518 PMC7745069

[13] Khan S. Cabanilla L. L., “Periodontal probing depth measurement: a review,” Compend. Contin. Educ. Dent. 30(1), 12–14 16, 18-21; quiz 22, 36 (2009). 19263761

[14] Larsen C. et al., “Probing pressure, a highly undervalued unit of measure in periodontal probing: a systematic review on its effect on probing pocket depth,” J. Clin. Periodontol. 36(4), 315–322 (2009). JCPEDZ 0303-6979 10.1111/j.1600-051X.2009.01383.x 19426178

[15] Biddle A. J. et al., “Comparison of the validity of periodontal probing measurements in smokers and non-smokers,” J. Clin. Periodontol. 28(8), 806–812 (2001). JCPEDZ 0303-6979 10.1034/j.1600-051X.2001.280813.x 11442742

[16] Chan H.-L. A. Kripfgans O. D., Dental Ultrasound in Periodontology and Implantology, Springer, Cham (2020).

[17] Chifor R. et al., “Three-dimensional periodontal investigations using a prototype handheld ultrasound scanner with spatial positioning reading sensor,” Med. Ultrason. 23(3), 297–304 (2021). 10.11152/mu-2837 33657191

[18] Tavelli L. et al., “Ultrasonographic tissue perfusion analysis at implant and palatal donor sites following soft tissue augmentation: a clinical pilot study,” J. Clin. Periodontol. 48(4), 602–614 (2021). JCPEDZ 0303-6979 10.1111/jcpe.13424 33465812 PMC8058327

[19] Holahan C. M. et al., “Effect of osteoporotic status on the survival of titanium dental implants,” Int. J. Oral Max Impl. 23(5), 905–910 (2008). 19014161

[20] Nguyen K. C. T. et al., “Localization of cementoenamel junction in intraoral ultrasonographs with machine learning,” J. Dent. 112, 103752 (2021). 10.1016/j.jdent.2021.103752 34314726

[r21] Nguyen K. C. T. et al., “High-resolution ultrasonic imaging of dento-periodontal tissues using a multi-element phased array system,” Ann. Biomed. Eng. 44(10), 2874–2886 (2016). ABMECF 0090-6964 10.1007/s10439-016-1634-2 27160674

[22] Nguyen K. C. T. et al., “Imaging the cemento-enamel junction using a 20-Mhz ultrasonic transducer,” Ultrasound Med. Biol. 42(1), 333–338 (2016). USMBA3 0301-5629 10.1016/j.ultrasmedbio.2015.09.012 26546266

[23] Das D. et al., “Another decade of photoacoustic imaging,” Phys. Med. Biol. 66(5), 05TR01 (2021). PHMBA7 0031-9155 10.1088/1361-6560/abd669 33361580

[24] Agrawal S. et al., “Light-emitting-diode-based multispectral photoacoustic computed tomography system,” Sensors 19(2), C2 (2019). SNSRES 0746-9462 10.1109/JSEN.2018.2879238 PMC6891584 31717260

[25] Moore C. et al., “Photoacoustic imaging for monitoring periodontal health: a first human study,” Photoacoustics 12, 67–74 (2018). 10.1016/j.pacs.2018.10.005 30450281 PMC6226559

[26] Lin C. Y. et al., “Photoacoustic imaging for noninvasive periodontal probing depth measurements,” J. Dent. Res. 97(1), 23–30 (2018). JDREAF 0022-0345 10.1177/0022034517729820 28880116 PMC5755810

[27] Giovannini M. Ardizzone S., “Anorectal ultrasound for neoplastic and inflammatory lesions,” Best Pract. Res. Cl Ga 20(1), 113–135 (2006). 10.1016/j.bpg.2005.09.005 16473804

[28] Casalegno J. S. et al., “High risk HPV contamination of endocavity vaginal ultrasound probes: an underestimated route of nosocomial infection?” PLoS One 7(10), e48137 (2012). POLNCL 1932-6203 10.1371/journal.pone.0048137 23110191 PMC3480505

[29] Berber E. Siperstein A. E., “Laparoscopic ultrasound,” Surg. Clin. N. Am. 84(4), 1061 (2004). 10.1016/j.suc.2004.05.005 15261753

[30] Sinjab K. et al., “Ultrasonographic evaluation of edentulous crestal bone topography: a proof-of-principle retrospective study,” Oral. Surg. Oral Med. Oral Pathol. Oral Radiol 133(1), 110–117 (2022). 10.1016/j.oooo.2021.07.006 34511351 PMC8688229

[31] Tattan M. et al., “Ultrasonography for chairside evaluation of periodontal structures: a pilot study,” J. Periodontol. 91(7), 890–899 (2020). 10.1002/JPER.19-0342 31837020

[32] Tsui B. C., “Ultrasound imaging to localize foramina for superficial trigeminal nerve block,” Can. J. Anaesth. 56(9), 704–706 (2009). CJOAEP 10.1007/s12630-009-9129-3 19504162

[33] Arconada-Alvarez S. J. et al., “The development and characterization of a novel yet simple 3D printed tool to facilitate phantom imaging of photoacoustic contrast agents,” Photoacoustics 5, 17–24 (2017). 10.1016/j.pacs.2017.02.001 28239554 PMC5314822

[34] Papapanou P. N. et al., “Periodontitis: consensus report of workgroup 2 of the 2017 world workshop on the classification of periodontal and peri-implant diseases and conditions,” J. Clin. Periodontol. 45(Suppl. 20), S162–S170 (2018). JCPEDZ 0303-6979 10.1111/jcpe.12946 29926490

[35] Schroeder A. B. et al., “The ImageJ ecosystem: open-source software for image visualization, processing, and analysis,” Protein Sci. 30(1), 234–249 (2021). PRCIEI 0961-8368 10.1002/pro.3993 33166005 PMC7737784

[36] Hallgren K. A., “Computing inter-rater reliability for observational data: an overview and tutorial (vol 8, pg 23, 2012),” Tutor Quant Methods 9(2), 95–95 (2013). 10.20982/tqmp.09.2.p095 PMC3402032 22833776

[37] Shrout P. E. Fleiss J. L., “Intraclass correlations: uses in assessing rater reliability,” Psychol Bull 86(2), 420–428 (1979). 10.1037/0033-2909.86.2.420 18839484

[38] Li X. et al., “Intravascular photoacoustic imaging at 35 and 80 MHz,” J. Biomed. Opt. 17(10), 1060051 (2012). JBOPFO 1083-3668 10.1117/1.JBO.17.10.106005 PMC3461096 23224004

[39] Wang S. et al., “The miniature pig: a useful large animal model for dental and orofacial research,” Oral Dis. 13(6), 530–537 (2007). 10.1111/j.1601-0825.2006.01337.x 17944668

[40] Giavarina D., “Understanding Bland Altman analysis,” Biochem. Med. 25(2), 141–151 (2015). 10.11613/BM.2015.015 PMC4470095 26110027

[41] Fjaertoft M. Johannessen A. C. Heyeraas K. J., “Micropuncture measurements of interstitial fluid pressure in normal and inflamed gingiva in rats,” J. Periodont. Res. 27(5), 534–538 (1992). 10.1111/j.1600-0765.1992.tb01828.x 1403583

[42] Isaia F. et al., “The root coverage esthetic score: intra-examiner reliability among dental students and dental faculty,” J. Periodontol. 89(7), 833–839 (2018). 10.1002/JPER.17-0556 29630720

